# Correlation and significance of COX-2, Ki67, VEGF and other immune indexes with the growth of malignant pulmonary nodules

**DOI:** 10.1186/s13019-022-02039-7

**Published:** 2022-11-16

**Authors:** Haoxin Guo, Wenfei Xue, Qingtao Zhao, Huanfen Zhao, Zhonghui Hu, Xiaopeng Zhang, Guochen Duan

**Affiliations:** 1 Hebei Medical University, Shijiazhuang, Hebei Province People’s Republic of China; 2 Department of Thoracic Surgery, Hebei General Hospital, 348, West He-Ping Road, Shijiazhuang, 050051 Hebei Province People’s Republic of China; 3 Children’s Hospital of Hebei Province, Shijiazhuang, Hebei Province People’s Republic of China

**Keywords:** Pulmonary nodules, Immunohistochemistry, Cyclooxygenase-2, Ki67, Vascular endothelial growth factor

## Abstract

**Objective:**

This study intends to explore the factors affecting the growth of pulmonary nodules in the natural process by immunohistochemical method.

**Methods:**

40 cases of pulmonary nodules followed up for more than 3 years were divided into growth group (n = 20) and stable group (n = 20). The expressions of cyclooxygenase-2 (COX-2), Ki67, vascular endothelial growth factor (VEGF), CD44V6, epidermal growth factor receptor (EGFR), double microsome 2 (MDM2) and transforming growth factor (TGF)-β1 in pulmonary nodules were detected by immunohistochemical method so as to explore the relationship between it and the growth of pulmonary nodules.

**Results:**

Compared with stable pulmonary nodules, the positive rates of COX-2, Ki67 and VEGF in the growth group were 85%, 80% and 55%, respectively. There was significant difference between the stable group and the growth group (P < 0.05). The correlation between other indexes and the growth of pulmonary nodules was not statistically significant (P_cd44v6_ = 0.104;P_EGFR_ = 0.337; P_MDM2_ = 0.49; P_TGF-β1_ = 0.141). In the subgroup of patients with non-invasive lung cancer, there was a correlation between VEGF and the growth of pulmonary nodules (P < 0.05).

**Conclusion:**

The high expression of COX-2, Ki67 and VEGF proteins may be significantly related to the growth of pulmonary nodules, and VEGF may be an important factor affecting the growth of malignant pulmonary nodules. This study intends to provide a research direction for further searching for the essential causes of the growth of pulmonary nodules.

## Introduction

With the popularization of lung cancer screening, a large number of patients with pulmonary nodules have been found. The National Comprehensive Cancer Network (NCCN) guidelines and American Cancer Society guidelines [1, 2] provide follow-up strategies for these patients. Annual follow-up may become an indispensable part of these patients' lives, and some even require 3-month follow-up. According to the guidelines, the size of nodules and the growth of solid components are the key to decide the next step of treatment. Persistent and stable nodules can continue to be observed, and increased solidity or larger diameter often suggest that nodules need surgical treatment. As a consequence, the degree of nodule growth indicated by chest CT directly determines the overall treatment strategy of patients with pulmonary nodules, so it is particularly important for clinicians to look for reasons for nodule growth or factors that can predict nodule growth.

At present, the research on predicting the growth of lung cancer is mainly based on the imaging findings to predict the growth trend of tumors [3, 4]. However, the biological indicators or testing methods that can be adopted for the growth of early lung cancer, that is, malignant pulmonary nodules, have not been systematically expounded and proved. Many studies have found that the essential factor of nodular growth is the unlimited expansion and invasion of tumor [5, 6]. Studies at home and abroad [7–15] have found that cyclooxygenase-2 (COX-2), Ki67, vascular endothelial growth factor (VEGF), CD44V6, epidermal growth factor receptor (EGFR), double microsome 2 (MDM2) and transforming growth factor TGF-β1 have certain clinical significance in tumor growth. However, the role of these immune indexes in the early stage of tumor, that is, the growth of malignant pulmonary nodules, has not been studied. The above immune indexes of malignant pulmonary nodules were analyzed to explore the correlation between each index and the growth of pulmonary nodules, and then to find the internal mechanism of nodule growth.

## Materials and methods

### General information

**Fig. 1:**
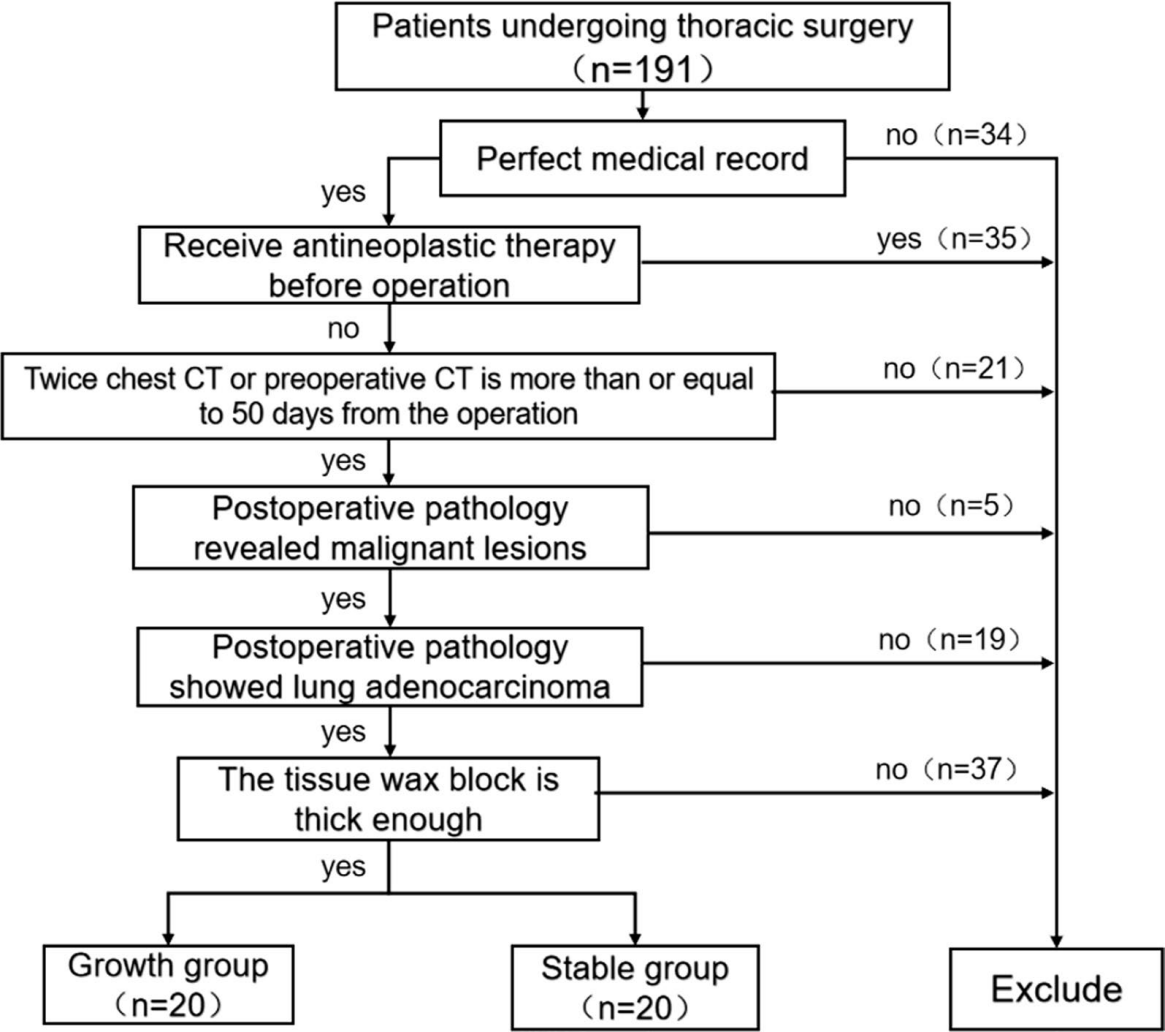
Inclusion and exclusion criteria and grouping flow chart (n: number of patients)

This research is a retrospective study. The patients who were followed up for at least 3 years in the Department of Thoracic surgery of Hebei Provincial People's Hospital from September 2016 to January 2021 found that the pulmonary nodules were significantly increased or stable, and finally underwent thoracoscopic minimally invasive resection and pathologically proved malignant patients. After screening, 40 patients were divided into groups (Fig. 1). Paraffin-embedded tissue sections were obtained, and the expression of antibodies in tissues was determined by immunohistochemical study. The clinical features such as gender, age and tumor size were collected.

All the patients involved in this study were frozen during the operation to determine the nature of the lesions, and specific operations were performed according to the diagnosis and treatment guidelines. See Table 1 for specific operation methods.

**Table 1:** Surgical methods for patients

	Growth group (n = 20)	Stable group (n = 20)
Wedge resection of lung	7	13
Segmental pneumonectomy	6	2
Lobectomy	7	5

*n* the number of patients

### Inclusion and exclusion criteria

#### Inclusion criteria

(1) Patients who received surgical treatment in the Department of Thoracic surgery of Hebei people's Hospital; (2) Two consecutive preoperative examinations were performed in our hospital with thin chest CT indicating pulmonary nodules. (3) In the stable group, the interval of chest CT was at least more than 3 years before operation, while in the growing group, the diameter of nodules increased or the solid components increased significantly in patients with CT ≥ 50 days. (4) All patients did not receive anti-tumor therapy such as chemotherapy and radiotherapy before operation. (5) Patients with complete clinical data; (6) Adenocarcinoma was confirmed by postoperative pathology (completed by two pathologists independently); (7) Paraffin paraffin blocks intact.

#### Exclusion criteria

(1). Preoperative chest CT results showed poor imaging quality; (2). Metastasis of nodules was found; (3). The case data were not perfect; (4). Postoperative pathological results showed benign patients; (5). Tissue wax was too thin.

#### Grouping standard

(A) Growth group: At least two deputy chief physicians of the imaging department read the CT, making it clear that the nodule diameter increased ≥ 3 mm or the tumor diameter increased by at least 20%, and the solid components increased significantly. (b) Stable group: Preoperative examination of chest CT and re-examination of CT after observation or measurement of the actual diameter of nodules after surgical resection showed that there was no growth of nodules or no increase in solid components or no increase in nodule volume and mass.

### Immunohistochemical method

1. Slicing dewaxing to water;
2. Putting the slices into EDTA repair solution (pH8.0), repairing 5 min under high pressure (timing from jet), and cooling naturally to room temperature;
3. Washing PBS for 3 times with 5 min each time;
4. Adding 3% hydrogen peroxide and incubate at room temperature for 20 min.
5. Washing PBS for 3 times with 5 min each time;
6. Sealing with sheep serum (working solution) at room temperature for 30 min.
7. The first antibody added on the slice stayed overnight at 4 ℃. After the slice was removed the next day, after the slice was restored to room temperature, it was washed in PBS for 3 times with 5 min each time.
8. Adding the second antibody to the slice and washing it in PBS for 5 min each time after avoiding light at 37 ℃ for 20 min.
9. DAB chromogenic solution shows positive staining, and tap water washing terminates coloration.
10. Hematoxylin re-dyeing, gradient alcohol dehydration, xylene transparency, neutral gum seal;
11. Observing under a microscope and taking pictures.

### Observation index

- The levels of COX-2, Ki67, VEGF, CD44V6, EGFR, MDM2 and TGF-β1 protein in nodular growth group and stable group were observed.
- The correlation between the increase of nodule diameter and the expression level of these proteins was analyzed.

### Statistical analysis

The statistical software SPSS24.0 was adopted to process the data, and the counting data were expressed as n (%). The correlation between the expression of COX-2, Ki67, VEGF, CD44V6, EGFR, MDM2, TGF-β1 and nodule growth was analyzed by Wilcoxon rank sum test, and the effect of COX-2, Ki67 and VEGF expression on lung nodule growth was analyzed by ROC curve. P < 0.05 indicated that the difference was statistically significant.

## Results

**Fig. 2:**
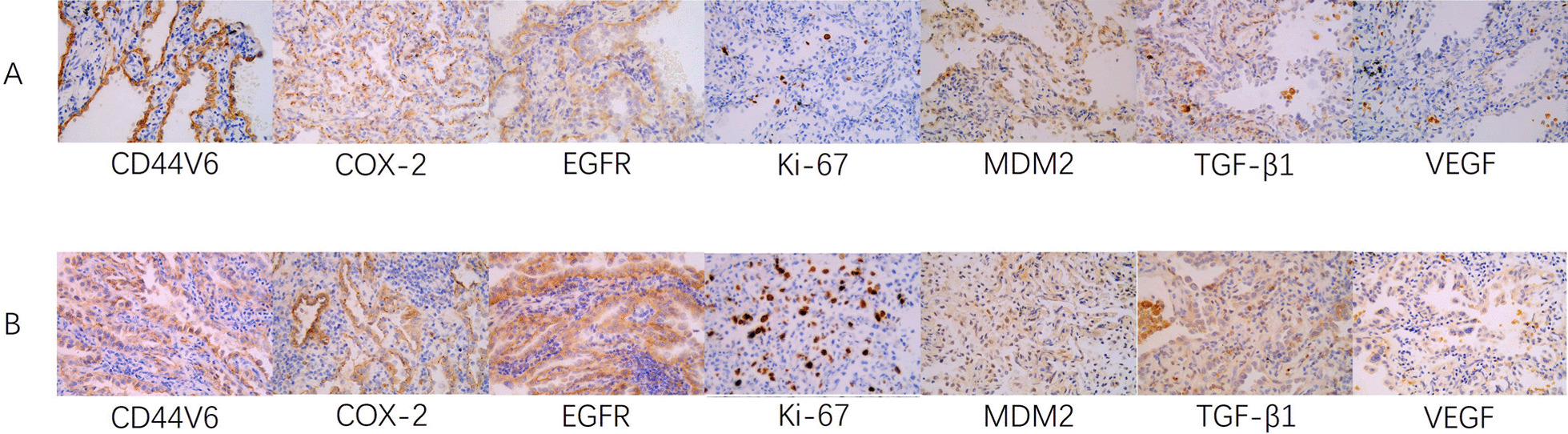
Immunohistochemical staining of groups A and B **A** stable group (×400); **B** growth group (×400)

40 patients with non-small cell lung cancer (NSCLC) were divided into two groups: the growth group (n = 20) and the stable group (n = 20). The basic clinical information of the patients in the corresponding group was analyzed (Table 2). The expressions of COX-2, Ki67, VEGF, CD44V6, EGFR, MDM2 and TGF-β1 in NSCLC tissues were detected by immunohistochemical analysis. Immunostaining of each index was observed in the nucleus and cytoplasm of cancer cells (Fig. 2). According to the results of immunostaining, the expression of each immune index was divided into four levels, which were negative, weak positive, moderate positive and strong positive respectively (Table 2). Before the statistical analysis of the results of various immune indicators, we first analyzed the correlation of basic clinical information of patients in the group from the aspects of age, gender, smoking history, tumor history, tumor stage, tumor invasiveness and so on. It was proved that there were intergroup differences between the growth of pulmonary nodules and tumor stage and tumor invasiveness (P stage = 0.000 and P invasiveness = 0.008), suggesting that there may be a correlation. There was no significant difference between the growth of nodules and age gender smoking history and tumor history (P > 0.05).

**Table 2:** Basic clinical information of patients

Indexes	Growth group (n = 20)	Stable group (n = 20)	P
Age (years)	66.5 ± 15.5	51.5 ± 17.5	0.056
Gender (male/female)	11/9	8/12	0.264
Smoking	7	5	0.366
*Stage*			
Tis	0	10	0.000
Stage I	20	10	
*Invasive*			
Non-infiltrating	11	19	0.008
Infiltrating	9	1	

*n* number of patients

The expressions of COX-2, Ki67, VEGF, CD44V6, EGFR, MDM2 and TGF-β1 in 40 patients were statistically analyzed (Table 3). It was found that the expression of COX-2, Ki67 and VEGF was correlated with the growth of early pulmonary nodules (P_COX-2_ = 0.023; P_Ki67_ = 0.036; P_VEGF_ = 0.026). However, there was no significant correlation between CD44V6, EGFR, MDM2, TGF-β1 and the growth of early pulmonary nodules.

**Table 3:** Analysis of the correlation between immune indexes and the growth of pulmonary nodules

	Growth group (n)	Stable group (n)	P
*cd44v6*			
−	6	5	0.104
+	8	2	
++	4	8	
+++	2	5	
*Cox-2*			
−	5	3	0.023
+	9	3	
++	4	7	
+++	2	7	
*EGFR*			
−	6	3	0.337
+	2	4	
++	6	4	
+++	6	9	
*Ki67*			
−	7	4	0.036
+	13	10	
++	0	2	
+++	0	4	
*MDM2*			
−	10	9	0.49
+	8	6	
++	1	3	
+++	1	2	
*VEGF*			
−	16	9	0.026
+	2	5	
++	2	6	
*TGF-β1*			
−	4	3	0.141
+	8	5	
++	7	7	
+++	1	5	

*n* number of patients

**Fig. 3:**
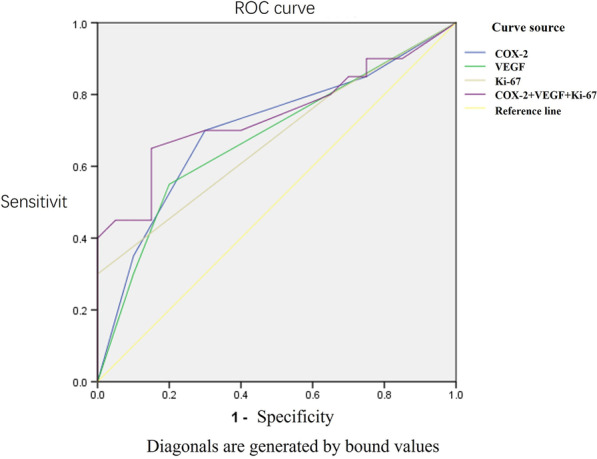
ROC curve

The ROC curves of COX-2, Ki67 and VEGF are drawn and analyzed (Fig. 3; Table 4). The results showed that the offline area of COX-2 was 0.703 (P = 0.028), which was statistically significant. It indicated that the expression of COX-2 was positively correlated with the growth of early pulmonary nodules. In addition, the offline area of COX-2 + Ki67 + VEGF was 0.741 (P = 0.009), which was statistically significant. It proved that the growth of early pulmonary nodules was more obvious when COX-2, Ki67 and VEGF were highly expressed.

**Table 4:** The area below the ROC curve

Test result variable	Area	Standard error^a^	Asymptotic significance^b^	Asymptotic 95% confidence interval	
				Lower limit	Upper limit
COX-2	0.703	0.085	0.028	0.536	0.869
VEGF	0.678	0.086	0.055	0.508	0.847
Ki67	0.673	0.086	0.062	0.504	0.841
COX-2 + VEGF + Ki67	0.741	0.081	0.009	0.582	0.901

The test result variable COX-2, VEGF, Ki67, COX-2 + VEGF + Ki67 has at least one binding value between the positive actual state group and the negative actual state group. There may be a deviation in the statistics

^a^According to the nonparametric assumption; ^b^Original hypothesis: true are = 0.5

This study further analyzed the correlation between each index and growth group and stable group in stage I lung cancer patients and non-invasive lung cancer patients (Tables 5, 6). We found that there was no significant correlation among immune indexes in stage I patients, but VEGF was statistically significant in non-invasive lung cancer patients subgroup (P_VEGF_ < 0.05). It is suggested that there is a correlation between VEGF and the growth of pulmonary nodules in this subgroup.

**Table 5:** Correlation analysis of each index and grouping in patients with stage I lung cancer

	Growth group (n)	Stable group (n)	P
*COX-2*			
−	3	0	0.198
+	3	6	
++	7	3	
+++	7	7	
*Ki-67*			
−	4	1	0.384
+	10	9	
++	2	0	
+++	4	0	
*VEGF*			
−	9	7	0.264
+	5	1	
++	6	2	
*CD44V6*			
−	5	0	0.397
+	2	7	
++	8	2	
+++	5	1	
*EGFR*			
−	3	0	0.575
+	4	1	
++	4	5	
+++	9	4	
*MDM-2*			
−	9	4	0.795
+	6	5	
++	3	1	
+++	2	0	
*TGF-β1*			
−	3	0	0.889
+	5	3	
++	7	6	
+++	5	1	

*n* the number of patients, *p* asymptotic significance

**Table 6:** Correlation analysis of each index and grouping in patients with non-invasive lung cancer

	Growth group (n)	Stable group (n)	P
*COX-2*			
−	2	5	0.089
+	2	9	
++	3	3	
+++	4	2	
*Ki-67*			
−	3	7	0.06
+	3	12	
++	2	0	
+++	3	0	
*VEGF*			
−	5	15	0.043
+	1	2	
++	5	2	
*CD44V6*			
−	4	6	0.337
+	0	7	
++	4	4	
+++	3	2	
*EGFR*			
−	2	6	0.368
+	1	2	
++	3	5	
+++	5	6	
*MDM-2*			
−	7	10	0.753
+	2	7	
++	1	1	
+++	1	1	
*TGF-β1*			
−	2	4	0.439
+	3	8	
++	5	6	
+++	1	1	

*n* the number of patients, *p* asymptotic significance

In addition, some other examination methods also have certain research value, such as imaging findings, tumor markers, etc. Binary logistic analysis was performed for some imaging findings (Table 7). As can be seen from the results in the table, there was no statistically significant correlation between tumor growth and imaging findings (P > 0.05). We then analyzed the results of the four tumor markers by Fisher test (Table 8). The results also showed that there was no significant correlation between tumor markers and lung nodule growth (P > 0.05). These results may have been limited by the sample size. In the following study, we will expand the sample size and make more accurate evaluation.

**Table 7:** Correlation between imaging findings of pulmonary nodules and tumor growth

	Growth group (n)	Stable group (n)	P
Lobulation	13	7	0.068
Spiculation	9	3	0.328
Pleural indentation	6	5	0.878
Vocule sign	9	6	0.567
*Proportion of solid components*			
< 0.25	4	8	0.931
> 0.25 and < 0.5	7	8	
> 0.5	9	4	

*n* the number of patients, *p* asymptotic significance

**Table 8:** Correlation between tumor markers and tumor growth

	Stable group (n = 20)	Growth group (n = 20)	P
CEA	3.08 ± 2.05	5.24 ± 4.11	0.605
SCC	1.38 ± 0.81	1.56 ± 1.02	0.205
NSE	13.89 ± 5.53	10.79 ± 3.99	0.741
Cytokeratin-19-fragment	2.31 ± 1.25	3.74 ± 2.75	0.065

*n* the number of patients, *p* precision significance, *CEA* Carcinoembryonic antigen, *SCCA* squamous cell carcinoma antigen, *NSE* neuron specific enolase

## Discussion

At present, most studies are based on imaging findings to predict the growth trend of tumors. For example, Hyun Jung Yoon et al. divided the patients into two groups according to the growth pattern of lung adenocarcinoma, extracted the CT imaging features reflecting marginal features and tumor doubling time and adopted to predict tumor doubling time [3]. YukihiroYoshida et al. also adopted high-resolution computed tomography images to predict the growth and changes of non-invasive adenocarcinoma [4]. However, the biological indicators or test methods that can be adopted to predict the growth of pulmonary nodules have not been systematically expounded and proved. However, there are also studies looking for factors related to nodule growth [16–18], which provide some research directions and ideas for predicting the growth of pulmonary nodules.

At present, a great quantity of studies [19–21] are based on the immunohistochemical analysis of middle and advanced lung cancer to find out the internal relationship between the relevant immune indexes, related factors and the occurrence, development, invasion, metastasis and prognosis of lung cancer. However, for the early lung cancer with ground glass nodules shown by imaging, there are few studies on tumor-related immune indexes. Some studies have proved that early detection of pulmonary nodules can effectively improve the survival rate of patients with lung cancer [22]. As a consequence, our research draws lessons from the study of immune indexes related to middle and advanced lung cancer, and selects immune indexes that promote tumorigenesis and development. It intends to observe the role of immune indexes in the growth or progression of early lung cancer, that is, pulmonary nodules. It intends to forecast the growth of pulmonary nodules to achieve the ultimate goal of early detection of malignant nodules, timely clinical treatment, and improve the survival rate of patients. The innovation of our research is to study the early lung cancer, that is, pulmonary nodules, and analyze the correlation between immune indexes and them. Through the statistical analysis of the experimental results, it is concluded that VEGF may be one of the important factors affecting early tumor progression.

VEGF plays an important part in the pathology of many cancer patients. Studies have shown that the increase of VEGF is significantly related to tumor proliferation, migration and endothelial cell invasion [10]. As a multi-subtype and multi-family protein first isolated from tumor cells, VEGF is a highly specific vascular endothelial growth factor. Its main role is to mediate angiogenesis in normal and diseased tissues, which is very important for tumor growth and is related to the poor prognosis of lung cancer [23]. The results of our study are basically consistent with the above point of view, suggesting that the high expression of VEGF is strongly related to the growth of lung cancer. It is speculated that the expression of VEGF plays a promoting role in the early growth of tumor, which may lay a foundation for further research on the internal factors of ground glass nodule growth.

Our study indicated that the patients with different expression levels of COX-2 in early lung cancer showed a high level of expression, the growth of pulmonary nodules was more significant. COX-2 is an enzyme which can converts arachidonic acid into prostaglandins (PGs). There is evidence that the overexpression of COX-2 in lung cancer promotes the proliferation, invasion, angiogenesis and anti-apoptosis of tumor cells [7]. Recently, more and more studies have focused on the discovery of transcription factors regulating the expression of COX-2, such as SP1, AP-2, CPSF4, CBP/P300, NF-kappa B and BPTF [24–29]. Although there are many regulatory factors, its carcinogenic effect is clear. In addition, the expression of COX-2 can regulate matrix metalloproteinases (MMP-7), leading to proliferation and invasion of lung adenocarcinoma [30]. The nodule growth in patients with pulmonary nodules is closely related to the expression of COX-2. Combined with the experimental results of our study, it can be further inferred that the expression of COX-2 can affect the growth velocity of pulmonary nodules in the early stage of lung cancer. The mechanism of COX-2 promoting the growth of early pulmonary nodules may be established, but it still needs to be proved by experiments, and the factors leading to these mechanisms are also worthy of further study.

Ki67 is a kind of macromolecular protein reflecting the activity of cell proliferation, and it is a widely adopted cell marker in the whole proliferative phase at present. It is expressed in every proliferation cycle except G0 phase. It is one of the best indicators of tumor active proliferation. It plays an important part in judging cell proliferation, analyzing cell growth and metastasis and predicting the prognosis of patients [9]. Our research shows that the increase of Ki67 can predict the growth trend of early pulmonary nodules. However, its correlation with nodular growth was weaker than that of COX-2. Related studies have also shown that Ki-67 can promote cell proliferation by regulating cell cycle [31–34]. However, the specific regulatory mechanism and the regulatory factors of Ki-67 itself are not very clear, which can be proved by experiments in follow-up studies.

However, the comprehensive consideration of COX-2, Ki67 and VEGF showed a strong correlation with nodular growth. Some studies have shown that COX-2 may be an active stimulator of VEGF in NSCLC tissues [35]. In addition, prostaglandin E2 (PGE2), the main product of tumor COX-2, regulates VEGF through EP3 and EP4, which has an important effect on tumor matrix formation and tumor growth, and regulates VEGF-C and promotes lymphangiogenesis in lung adenocarcinoma through EP1 [36, 37]. Combined with the above related studies, it is concluded that besides regulating VEGF, COX-2 has other regulatory pathways that can directly or indirectly affect tumor growth, but in the real process of tumor growth, there is still some uncertainty about which regulatory pathway is dominant, which may also be one of the reasons why the correlation between COX-2 and early lung cancer growth is more significant than VEGF in this study. In the early stage of pulmonary nodule growth, COX-2 and VEGF have been combined to promote nodule growth. In the study of the relationship between COX-2 and Ki-67, it was found that the average value of Ki-67 labeling index in COX-2 positive tumors was significantly higher than that in COX-2 negative tumors [38]. However, our study is based on patients with gastric cancer as the research object. There is no article to prove that there is a relationship between COX-2 and Ki-67 in the occurrence and development of lung cancer. Similarly, there are no reports about the interaction between VEGF and Ki-67 in the process of tumor growth. Combined with the results of our study, as a marker of tumor growth, the relationship between Ki-67 and COX-2 and VEGF may be due to the performance of COX-2 and VEGF promoting tumor growth, but it can not be ruled out that COX-2 or VEGF have some effects on Ki-67, which needs to be verified by follow-up experiments.

CD44v6 is a soluble intercellular adhesion molecule, which is closely related to the invasion, progression and metastasis of malignant tumors [11]. CD44s and CD44V6 were predominantly present in normal lung tissue [39, 40]. With the activation of receptors on the surface of CD44 cells, some downstream signal events will be expressed accordingly [41, 42]. This interaction can lead to tumor growth. The expression of CD44s and CD44v6 in the origin cells of lung adenocarcinoma is inconsistent [43]. Given the results that we got in our study, it can be concluded that there is no close relationship between CD44V6 and nodule growth in early lung nodules, which may be related to the different origin cells of lung adenocarcinoma. EGFR is a transmembrane receptor tyrosine kinase that regulates cell proliferation and growth-related signal transduction through phosphorylation [12]. At present, studies have shown that EGFR can promote cell division and proliferation, and up-regulated expression in many malignant tumors, including lung cancer [13]. However, regardless of the metastatic status, NSCLC had a higher amplification rate of EGFR gene, and the amplification rate of EGFR gene was related to the differentiation degree of NSCLC [44]. The subjects of this study are patients with early NSCLC, so it can be suspected that the amplification rate of EGFR gene is lower in patients with early pulmonary nodules. The content of MDM2 oncogene as a proto-oncogene in normal tissues is very small, but the positive expression rate of MDM2 oncogene in NSCLC tissues increases with the increase of histological differentiation, and is significantly different from lymph node metastasis and tumor TNM stage [14]. TGF-β1 is a complex cytokine, which is closely related to tumor. In the process of tumor cell proliferation, its regulatory role has two sides [15]. In the early stage of cancer, TGF-β inhibits the growth of tumor cells and induces tumor cell apoptosis. On the contrary, in the later stage of tumor progression, it acts as a promoter to promote tumor invasion and metastasis [45]. As an early stage of lung cancer, the role of TGF-β1 in lung nodules changes from growth inhibition to promotion with the progression of lung cancer, which may also make the correlation between it and the growth of lung nodules unclear. As a consequence, the above factors were not significantly correlated with the growth of early pulmonary nodules in this study.

Combined with our experimental results, postoperative immunohistochemical staining will have a positive impact on clinical treatment. For patients with pulmonary nodules with high expression of COX-2, Ki67 and VEGF, the growth of nodules is in a rapid growth state. For patients with inert pulmonary nodules whose immunohistochemical results showed that COX-2, Ki67 and VEGF were expressed at low levels after operation, the growth rate of nodules was relatively slow. In clinical practice, most patients can determine whether they need surgery through preoperative imaging and liquid examination. However, there are still a few patients whose preoperative examination can not determine whether they can be operated. For example, the imaging findings of patients before surgery suggest that the nodules are large, but the results of multiple examinations reflect that the growth of nodules is not obvious. When the patient is hesitant about the choice of treatment scheme due to consideration of other factors such as basic disease or physical condition, the immunohistochemical results can be obtained through puncture biopsy or tracheoscopy before operation to guide the selection of treatment scheme. According to the analysis of immunohistochemical results, if the expression of COX-2, Ki67 and VEGF is increased, it can be considered that the lung nodule growth rate of these patients is high. We will advise them to take surgical treatment in time. If the immunohistochemical results of patients suggest that the growth of nodules is slow, we can also follow up these patients regularly to observe the changes of pulmonary nodules.

This study can also provide the basis for genetic testing in the blood of patients in the follow-up study, and then screen the genotypes that promote the growth of nodules. We will find out the key factors that promote the growth of nodules through the analysis of gene expression. In the clinical environment, due to difficulties in operation or multiple complications of patients, some patients may not be able to obtain the expected immunohistochemical results through preoperative puncture biopsy or tracheoscopy. For this kind of patients, we can determine whether the patient has a gene that promotes nodule growth by testing before operation, so as to guide clinical treatment. The exploration of this aspect will be the focus of our research in the future.

This study has the following limitations: (1) The experiment in this paper is a retrospective analysis. Due to the failure to carry out the clinical experiment design in advance, our study can only analyze the existing clinical data. The immunohistochemical results are all independent tests after surgical resection of pulmonary nodules, and the relevant test results of patients before surgery are not obtained. Therefore, compared with prospective studies, our experimental results also have certain limitations. In the follow-up study, we will adopt a prospective experimental design. Patients who could obtain immunohistochemical results before operation and found pulmonary nodules for the first time were included, and their test data were obtained for clinical observation. (2) The overall sample is small, which may not fully reflect the situation of all patients. (3) This study only collected preoperative imaging parameters, no relevant hematological indicators, postoperative data, etc., may also affect the final results. (4) This study is limited to the study of the relationship between tumor-related immune indicators and the growth of pulmonary nodules, and will be further studied at the molecular level in the future. As a new type of index to predict the growth of pulmonary nodules, COX-2, Ki67 and VEGF still need to further explore the specific mechanism of their effects on the growth of pulmonary nodules. Large-scale multicenter, prospective clinical studies are needed to verify their clinical value.

## Conclusion

COX-2, Ki67 and VEGF are significantly correlated with the growth of early lung cancer. The correlation between VEGF and the growth of early lung cancer is the most obvious. These results provide research value and direction for further searching for the internal factors of the growth rate of early lung cancer.

## Data Availability

The datasets used and analysed during the current study are available from the corresponding author on reasonable request.

## Abbreviations

COX-2: Cyclooxygenase-2
VEGF: Vascular endothelial growth factor
EGFR: Epidermal growth factor receptor
MDM2: Double microsome 2
TGF: Transforming growth factor
NCCN: The National Comprehensive Cancer Network
NSCLC: Non-small cell lung cancer
PGs: Prostaglandins
MMP-7: Matrix metalloproteinases
PGE2: Prostaglandin E2
